# Workplace and social support, treatment satisfaction, and their impact on quality of life in Swedish women with multiple sclerosis: a cross-sectional survey study

**DOI:** 10.1136/bmjopen-2024-087563

**Published:** 2024-12-20

**Authors:** Alejandra Machado, Elin Wredendal, Katharina Fink, Emilie Friberg

**Affiliations:** 1 Division of Insurance Medicine, Department of Clinical Neuroscience, Karolinska Institutet, SE-171 77, Stockholm, Sweden; 2 Bristol Myers Squibb, Stockholm, Sweden; 3 Division of Neurology, Department of Clinical Neuroscience, Karolinska Institutet, SE-171 77, Stockholm, Sweden

**Keywords:** Multiple sclerosis, Health, Work Satisfaction, Quality of Life, Perceived Social Support, Patient Satisfaction

## Abstract

**Abstract:**

**Objective:**

To evaluate health-related quality of life (HRQoL), in relation to support from work, social, as well as treatment satisfaction, in women with multiple sclerosis (MS). Further, to predict the satisfaction on these support dimensions based on sociodemographic and clinical variables.

**Design:**

Cross-sectional survey: a web-based questionnaire conducted in 2021 of people with MS (PwMS) linked to Nationwide Swedish registers.

**Setting:**

Sweden.

**Participants:**

Working women with MS, living in Sweden, aged 20–50 responding to the survey during the spring of 2021 (n=2967).

**Outcome measures:**

Health-related quality of life was measured using the EuroQol Visual Analogue Scale (EQ-VAS). Linear regression models were applied to estimate the association between demographic and clinical factors, as well as reported survey answers with HRQoL. Odds of perceived satisfactory support in one, two or all three support dimensions (work, social or treatment) were performed with multinomial logistic regressions.

**Results:**

Lower MS severity and fatigue, higher cognitive processing speed, living in cities and higher educational attainment were individually associated with higher levels of HRQoL (p<0.001). Contrary, lower HRQoL was associated with progressive type of MS, self-reported visible or invisible symptoms, and no or unsatisfactory support from work, social and treatment (p<0.001). When explored altogether, higher levels in HRQoL were predominantly explained by lower MS severity (t=−9.318, p<0.001), less fatigue (t=−22.190, p<0.001) and more support from work (t=4.824, p<0.001) and to some extent, social support (t=−2.448, p=0.014). Further, compared with women who reported no support, those experiencing lower fatigue and receiving ongoing treatment were more likely to receive support in one or more of all three dimensions (work, social and satisfaction with their treatment). In contrast, higher HRQoL (OR=1.033; CI=1.015 to 1.052) was only significant when receiving simultaneous support from all three support dimensions.

**Conclusion:**

Clinical factors and support from work and social support are the strongest contributors to HRQoL in working women with MS. Further, support across several life dimensions is essential when assessing HRQoL. Particularly, satisfaction with the perceived support from work, which plays a crucial role in the HRQoL of women with MS. This underscores the importance of prioritising clinical management and strong support systems to significantly improve HRQoL outcomes in patients with MS.

**STRENGTHS AND LIMITATIONS OF THIS STUDY**

- The study integrates data from the Swedish MS Registry, which covers 87% of Sweden’s population with multiple sclerosis (MS), with a 71.4% survey response rate from women, offering a comprehensive overview of individuals with MS in Sweden.
- Despite a high response rate among women, a minority of women did not complete the survey, potentially introducing non-response bias into the findings.
- The cross-sectional design of the study limits our ability to a cause-and-effect relationship, meaning we cannot conclude that perceived support directly impacts health-related quality of life for people with MS.
- People with MS may receive support but this does not automatically ensure it is perceived as supportive.

## Introduction

 Multiple sclerosis (MS) is a chronic and potentially disabling neurological disease, typically diagnosed between the ages of 20 and 40.^1^ Women are two to three times more likely to be diagnosed with MS compared with men.^1^ MS can manifest with a wide range of cognitive, mental and physical symptoms, with fatigue being one of the most prevalent and burdensome in the patient’s daily life.^2^ As follows, women with MS must adapt to and oversee this array of symptoms that can limit engagement across various aspects of life, including work, having a family and social interactions.^3 4^ Further, the impact of parenthood on the work capacity of women with MS is more pronounced compared with men,^5^ and sustaining employment is positively associated with better health-related quality of life (HRQoL).^6^

One of the most common measures to assess HRQoL in MS is the EuroQol five-dimensional questionnaire (EQ-5D).^7^ People with MS (PwMS) report lower levels of HRQoL when compared with the general population.^8 9^ Specifically, domains like work, social life, relationships, housework, mood, fatigue, cognitive processing speed, walking and balance have been identified as relevant in quality of life in MS.^10^ Indeed, both fatigue and cognitive difficulties, prevalent MS symptoms regardless of disability levels,^4 11 12^ have demonstrated a major detrimental effect on HRQoL of PwMS.^13^ Further, increased disability is known to have a direct impact on HRQoL.^9 13^ However, women seem to report less impact of MS on HRQoL as compared with men.^9^ Regardless, patients with MS have demonstrated improved results in their HRQoL when receiving support from their workplace, as well as social support.^14 15^ Since employment is positively linked to the overall well-being of PwMS,^16^ we were particularly interested in exploring workplace support alongside support from family and friends, hereby defined as social support. In addition, recent advancements in MS treatment, which focus on reducing disability progression, have demonstrated a direct positive impact on the quality of life for PwMS.^17^ Accordingly, treatment satisfaction has been associated with higher HRQoL or an improvement in HRQoL.^18^

To the best of our knowledge, no other study has explored HRQoL with the perceived support from different life dimensions (work, social and treatment) among working women with MS. Using data from the largest cross-sectional survey of patients with MS conducted in Sweden to date, we aim to explore the relationship of sociodemographic and clinical characteristics, and self-reported support from work, social support and treatment with HRQoL among aged-working women. Second, we aim to explore which factors best predict satisfaction with perceived support in any or all three life dimensions.

## Methods

A cross-sectional study was conducted using a web-based survey answered by PwMS related to their health, work and perceived support, as well as individual-level linked register data.

### Data sources and study population

Individuals listed in the Swedish MS Registry (SMSreg),^19^ with ages between 20 and 50 years, and living in Sweden were invited to answer a web questionnaire between May and September 2021. The survey was sent out to 8458 PwMS, with a response rate of 52% (4412 participants), with a higher response rate among women (71.4%; n=3148), the sample included in this study. Further details of the study population are published elsewhere.^4^ Statistics Sweden administered the survey and linked the responses to individual-level clinical data from the SMSreg and demographic data from Statistics Sweden.^20^ The anonymised data was then delivered to the researchers.

Following, exclusion criteria were applied if: (1) reporting ‘no occupation’ (n=415), (2) not actively working (n=26) or (3) unavailable information on self-reported health (n=10). The final study population consisted of 2967 working women (see online supplemental figure 1 for further details for criteria application).

### Register variables

The long integrated database for health insurance and labour market studies (LISA), held by Statistics Sweden, was used to obtain socio-demographic variables (sex, educational level, country of birth, marital status, living with children at home and type of living area). Age was calculated as the difference between the day of birth and the date of survey response. Additionally, the following clinical variables were obtained from the SMSreg:

- Type of MS (relapsing-remitting, secondary-progressive, primary-progressive, missing).
- Time since diagnosis, calculated as the difference, in years, between the date of MS diagnosis and the date of survey response.
- MS severity was assessed with the most recently available Expanded Disability Status Scale (EDSS) score^12^ and occurring within 3 years of the survey response (n=257 set to missing) and participants with secondary-progressive or primary-progressive MS with an EDSS score of 0 were set to missing (n=9).^21^ The scores were then categorised (as mild (EDSS=0–2.5), moderate (3–5.5) and severe (6–9.5) or missing).
- Cognitive processing speed assessed with the Symbol Digit Modalities Test (SDMT) and occurring within 3 years of the survey response (n=347 set to missing).
- Ongoing treatment at the time of survey (yes, no).

### Survey measures

Several measures were selected from the questionnaire. Specifically, closed-ended questions related to self-rated health (EQ-5D-3L and EuroQol Visual Analogue Scale (EQ-VAS)), fatigue, depression and anxiety subscales (Neuro-QoL), disclosure and perceived support. In addition, an open-ended question was included related to reporting MS restrictions. Responses from this open-ended question ‘Which MS symptom do you experience as the most limiting?’ informed on the most limiting symptom and were grouped into three categories following the European and American MS guidelines for symptom management, as reported elsewhere^4 21^:

- No symptom*.*
- Visible symptoms (movement/ambulation problems, vision problems, bladder, bowel and sexual dysfunction and dysphonia, dysarthria and dysphagia).
- Invisible symptoms (fatigue and cognitive difficulties, sensory and pain, mood problems/depression, other—medication-related symptoms, unspecified symptoms or other psychosocial implications of MS symptoms).

Disclosure at work was explored with the following question: ‘Have you told your boss about your MS diagnosis?’ with four possible answers: Yes; No, but I will; No; Not applicable.

Further, perceived support from work or social support was investigated with the following statements: ‘I receive the support I would like from my closest boss’ or ‘I receive the support I would like from my friends and family’. Both questions could be answered with five options: Agree completely; Agree to some extent; Do not agree at all; Not applicable; Have not told.

Finally, participants were asked ‘What treatment/s do you currently have for your MS?’ followed by a closed-end question to explore their satisfaction with the current treatment: ‘Are you satisfied with your treatment?’ with three possible answers: Yes; Partly; No. However, only the latter question was used in the study, as information about ongoing treatment was based on the registered data (‘ongoing treatment’).

The web-based survey has been translated into English and is included as an Annex in the online supplemental material.

### Outcome measure

The EQ-VAS was used to determine the self-rated health among the actively working women with MS. It derives from the EQ-5D, which assesses mobility, self-care, usual activities, pain/discomfort and anxiety/depression. The EQ-VAS form, however, is a vertical thermometer-like scale ranging from 0 (worst imaginable health) to 100 (best imaginable health), where respondents rate their overall health status. EQ-VAS scores have shown consistency with the severity of health states from the EQ-5D-3L.^22^

### Statistical analyses

Descriptive statistics was used to characterise the study population using frequencies and proportions for all categorical variables and mean and SD for continuous variables, after confirming normal distribution. Comparisons between categorical or continuous variables were performed using χ^2^ and analysis of variances, respectively, using Benjamini–Hochberg correction for multiple comparisons.

Simple linear regressions were applied to estimate the association between all sociodemographic and clinical variables, as well as other answers from the survey (limiting symptoms and support from work, social and treatment), with the health-related quality of Life (EQ-VAS). All variables were examined to ensure they met the necessary assumptions for multiple linear regression analysis. Certain variables (depression and anxiety subscales) were excluded after introducing multicollinearity issues. Categorical variables with more than two levels were converted into dummy variables, using one of the levels as a reference (constant in the model). Missing data was not included in the analyses. Subsequently, variables which individually showed a significative association with HRQoL were then included in a multiple linear regression with a backward elimination method. Additional analyses (not shown) were performed to confirm that the selection of variables included in the model contained the highest proportion of variance (R^2^) and adjusted variance (adj. R^2^) considering the number of predictors in the model.

Finally, the likelihood of reporting having support from none, any or all dimensions (based on ‘completely agree’ or ‘yes’ responses to support from work, family and treatment satisfaction), was estimated using multinomial logistic regression. Results are reported as OR with 95% CI. Furthermore, an additional sensitivity analysis was conducted, which involved participants who had disclosed their condition to their boss and could thus be queried about receiving support from their workplace.

All statistical analyses were performed using SPSS .27 and a p value of <0.05 was considered statistically significant.

### Patient and public involvement

None.

## Results

The average age of the study participants was 40.1 (table 1). The majority had a university educational level (68.9%), were born in Sweden (89.2%), were not married/not in partnership (53.5%), had children living at home (60.4%) and lived in cities or suburban areas (82.9%). Relapsing-remitting MS (RRMS) was the most common type of MS (94.4%). Most participants had an EDSS reflecting a low to mild MS severity (84.7%). Further, most women with MS were on treatment (90.8%) at the time of the survey, where 99.6% of these treatments were disease-modifying treatments (DMT). Available results for SDMT and fatigue scores reflected average scores, although the variance was wide-ranging (table 1).

**Table 1:** Sociodemographic and clinical characteristics of all working women included in the study

Sociodemographic characteristics	Women with MS(n=2697)	
	n (%)	M (SD)
Age		
20–29 years	257 (9.5)	40.1 (8.1)
30–39 years	897 (33.3)	
40–49 years	1384 (51.3)	
50–51 years	159 (5.9)	
Educational level		
Non-university	685 (31.1)	
University	1518 (68.9)	
Country of birth		
Sweden	2405 (89.2)	
Other	292 (10.8)	
Marital status		
Not married nor cohabitant/divorced/widowed	1444 (53.5)	
Married/cohabitant	1253 (46.5)	
Children <18 at home		
Yes	1628 (60.4)	
No	1069 (39.6)	
Type of living area		
Cities	1207 (44.8)	
Suburbs/towns	1028 (38.1)	
Rural areas	462 (17.1)	
**Clinical characteristics (register data)**	**n (%)**	**M (SD)**
Type of MS		
Relapsing remitting (RRMS)	2547 (94.4)	
Secondary progressive (SPMS)	96 (3.6)	
Primary progressive (PPMS)	31 (1.1)	
Missing	23 (0.9)	
Time since diagnosis		
0–4 years	746 (27.7)	8.8 (6.2)
5–9 years	731 (27.1)	
10–14 years	554 (20.5)	
15+ years	493 (18.3)	
Missing	173 (6.4)	
MS severity (EDSS)		
Mild (0–2,5)	1857 (84.7)	1.5 (1.4)
Moderate (3–5,5)	289 (13.1)	
Severe (4–9)	49 (2.2)	
Cognitive processing speed (SDMT)		
Available result	1928 (65.0)	58.1 (12.2)
(min.–max.)		(11–110)
Fatigue (Neuro-QoL)		
Available result	2691 (90.7)	50.4 (95.1)
(min.–max.)		(29.5–74.1)
Ongoing treatment		
Yes	2449 (90.8)	
Yes (of which DMTs)	2439 (99.6)	
No	248 (9.2)	

Note: some 50-year-old participants turned 51 by the time they conducted the survey.

DMTsdisease modifying treatmentsM, meanmax.maximummin.minimumMS, multiple sclerosis; n, sample size

Regarding the survey questions (table 2), the majority reported invisible symptoms (63.1%) as the most limiting symptom, had disclosed to their closest boss (77.2%) and were satisfied with support from work (62.2%), social support (62.8%) and treatment (79.3%).

**Table 2:** Number and proportion of responses to open-ended and closed-ended survey questions

	All women with MS(n=2697)
Most limiting symptom	
No symptom	326 (12.1)
Visible symptom	363 (13.5)
Invisible symptom	1703 (63.1)
No response	305 (11.3)
Disclosure: ‘Have you told your boss about your MS diagnosis?’	
Yes	2082 (77.2)
No, but I will; No; Not applicable; No response	615 (22.8)*
Support from work: ‘I receive the support I would like from my closest boss’	
Agree completely	1294 (48.0)
Agree to some extent	550 (20.4)
Do not agree at all	214 (7.9)
No response	24 (0.9)
Missing (did not disclose to their boss)	615 (22.8)*
Social support: ‘I receive the support I would like from my family and friends’	
Agree completely	1694 (62.8)
Agree to some extent	759 (28.1)
Do not agree at all	100 (3.7)
Not applicable/Have not told	126 (4.7)
No response	18 (0.7)
Treatment satisfaction: ‘Are you satisfied with your treatment?’	
Yes	2140 (79.3)
Partly	456 (16.9)
No	55 (2.0)
No response	47 (1.7)

Note: treatment satisfaction relates to the participant’s opinion regarding their treatment and should not be confused with ‘ongoing treatment’, which is data extracted from the clinical register regarding if the participant has a treatment or not.

* Participants who responded not having disclosed to their boss or not applicable or missing, where not enquired if receiving support from work (thus, they are set as missing). Hence, from those enquired (n=2082): aAgree completely (62.2%), Agree to some extent (26.4%), Do not agree (10.3%), or nNo response (1.2%).

MSmultiple sclerosis

Simple linear regressions between HRQoL and sociodemographic variables showed that only non-university level and living in cities were associated with lower and higher HRQoL, respectively (online supplemental table 1). In addition, a worse clinical profile (progressive types of MS, reporting visible and invisible symptoms, higher EDSS, lower cognitive processing speed and higher fatigue) was associated with worse HRQoL. However, when all key factors were considered, the previous sociodemographic associations with HRQoL disappeared. The remaining results were lower MS severity and fatigue along with satisfaction with support from work had a positive association with HRQoL and were associated with higher HRQoL (see table 3). Whereas partly perceived social support had a negative association with HRQoL. This multiple regression model explained 66% of the variance in HRQoL, with an adjusted R^2^ of 43%.

**Table 3:** Multiple regression association of health-related quality of life (HRQoL) with sociodemographic and clinical characteristics as well as self-reported health and perceived support responses from the survey (n=1309)

Explanatory variable	R	Adj. R^2^	Unstandardised coefficient		Standardised coefficient	T	P value
			B	SE	Beta		
(Constant)			118.964	2.790		42.642	**<0.001**
University education					0.015	0.687	0.493
Living in cities					0.017	0.837	0.403
MS severity (EDSS)			−2.359	0.253	−0.214	−9.318	**<0.001**
Cognitive processing speed (SDMT)			0.049	0.028	0.038	1.763	0.078
Fatigue (Neuro-QoL)			−0.895	0.040	−0.516	−22.190	**<0.001**
Limiting symptoms							
Visible symptom			−1.891	1.026	−0.041	−1.843	0.066
Invisible symptom					−0.013	−0.380	0.704
Support from work	0.437	0.434					
Agree completely			3.878	0.804	0.115	4.824	**<0.001**
Agree to some extent					0.030	0.271	0.787
Do not agree at all			2.371	1.248	0.044	1.900	0.058
Social support							
Agree completely					−0.002	−0.039	0.969
Agree to some extent			−1.888	0.771	−0.053	−2.448	**0.014**
Do not agree at all					−0.016	−0.735	0.462
Treatment satisfaction							
Partly					−0.017	−0.785	0.433
No					0.006	0.288	0.774

Note: Predictive variables included in the model were selected according to a significant result from the simple regression models (Supp. Material, online supplemental table 1Table 1). Backwards elimination method was performed in the multiple regression model with the results corresponding to the 9thninth model. The model was significant, *F*(7,196) = 144.029, pp<0.001, indicating more than one predictor significantly affects HRQoL. To avoid collinearity, the type of MS and time since diagnosis were excluded from this model owing to its association with EDSS (r=−0.308, pp<0.001; r=0.228, pp<0.001 and after posterior collinearity diagnostics >0.95). All levels of the categorical variables were dummied (0=no, 1=yes). References for categorical variables: limiting symptoms=No symptoms; Ssupport from work and Ssupport from family and friends=Not applicable or have not told; and treatment satisfaction variables=Yes. Bold values denote statistical significance at the p <0.05 level.

EDSSExpanded Disability Status ScaleHRQoLhealth-related quality of LifeMSmultiple sclerosisSDMTSymbol Digit Modality Test

Finally, a higher likelihood to report satisfaction with perceived support from work, social or treatment was associated with younger age, lower EDSS, higher cognitive processing speed and fewer MS restrictions (self-reported invisible and visible symptoms) (table 4). However, when adjusted by age, educational level and all clinical variables, only lower fatigue scores and being on treatment were associated with satisfaction with support from one life dimension, compared with those who reported no support. Similarly, satisfaction with support from two and all three dimensions was associated with lower fatigue, being on treatment and non-university educational level. Importantly, higher HRQoL was associated with higher odds of responding by having support from all three dimensions (work, social and treatment) compared with those who had no support. Descriptive characteristics for multinomial regressions can be found in online supplemental table 2. Additional sensitivity analysis repeating the adjusted multinomial logistic regression was performed for the subsample who had disclosed their MS at work with no changes in relation to previous results (online supplemental table 3).

**Table 4:** Unadjusted and adjusted multinomial logistic regression analysis for satisfied support (‘completely agree’ or ‘yes’) from work, social and/or treatment dimensions

	Unadjusted OR (95% CI)			Adjusted OR (95% CI)		
	Support in one dimension	Support in two dimensions	Support in all three dimensions	Support in one dimension	Support in two dimensions	Support in all three dimensions
Age group						
20–29	1.295 (0.650–2.580)	**2.132(1.110–4.094**)	**2.131(1.097–4.138**)	1.587 (0.527–4.780)	2.619 (0.896–7.659)	2.492 (0.839–7.404)
30–39	1.253 (0.876–1.792)	1.117 (0.789–1.583)	1.342 (0.942–1.913)	1.311 (0.775–2.217)	1.176 (0.699–1.979)	1.343 (0.789–2.288)
50+	0.799 (0.408–1.564)	0.870 (0.459–1.649)	1.202 (0.634–2.280)	1.190 (0.416–3.403)	1.234 (0.441–3.455)	1.448 (0.501–4.186)
Educational level						
Non-university	1.289 (0.895–1.857)	**1.418 (0.997–2.018**)	**1.549(1.083–2.216**)	1.392 (0.803–2.414)	**1.724(1.003–2.965**)	**2.062(1.185–3.588**)
Country of birth						
Not Sweden	1.142 (0.695–1.876)	0.953 (0.587–1.550)	0.704 (0.423–1.171)			
Children <18 at home						
No	0.729 (0.526–1.010)	0.882 (0.645–1.207)	0.876 (0.636–1.205)			
Marital status						
Not married nor cohabitant	0.859 (0.622–1.187)	0.977 (0.715–1.334)	0.867 (0.631–1.191)			
Type of living area						
Suburbs/towns	0.892 (0.626–1.271)	1.126 (0.801–1.583)	1.286 (0.908–1.820)			
Rural areas	0.1098 (0.692–1.743)	1.084 (0.691–1.701)	1.327 (0.841–2.096)			
MS severity (EDSS)	0.**851 (0.759–0.955**)	0.**816 (0.730–0.913**)	**0766 (0.681–0.860**)	0.935 (0.789–1.106)	1.045 (0.886–1.231)	1.026 (0.864–1.219)
Cognitive processing speed (SDMT)	1.010 (0.992–1.028)	**1.023(1.005–1.041**)	**1.029(1.011–1.048**)	0.999 (0.979–1.018)	1.012 (0.993–1.031)	1.013 (0.993–1.033)
Fatigue (Neuro-QoL)	**0**.**952 (0.934–0.970**)	**0.920(0.903–0.937)**	**0**.**900 (0.883–0.917**)	**0**.**961 (0.928–0.996**)	**0**.**930 (0.898–0.963**)	**0**.**919 (0.886–0.952**)
HRQoL (EQ-VAS)	**1.023(1.014–1.032**)	**1.038(1.030–1.047**)	**1.056(1.046–1.066**)	1.007 (0.990–1.024)	1.015 (0.999–1.032)	**1.033(1.015–1.052**)
Ongoing treatment						
No	**0.483(0.310–0.752)**	**0.450(0.294–0.687)**	**0.305(0.192–0.485)**	**0.368(0.173–0.785)**	**0.356(0.170–0.743)**	**0.201(0.088–0.461)**
Most limiting symptom						
Visible symptom	0.399 (0.168–0.948)	**0**.**344 (0.151–0.787**)	**0**.**274 (0.119–0.629**)	0.506 (0.129–1.996)	0.515 (0.135–1.968)	0.513 (0.133–1.981)
Invisible symptom	0.507 (0.236–1.091)	**0**.**286 (0.137–0.597**)	**0**.**233 (0.111–0.489**)	0.786 (0.219–2.822)	0.710 (0.203–2.488)	0.769 (0.218–2.709)

References (predictive variables): Aage (40–49 years), Eeducational level (University), Ccountry of birth (Sweden), Cchildren at home (yes); marital status (married or cohabitant), type of living area (cities), ongoing treatment (yes) and most limiting symptom (no symptom).

Reference (dependent variable): Nno Ssupport.

To note: sample sizes may variatey depending on available data, specifically for EDSS, SDMT and type of MS). Adjusted model samples size n=1689. Bold values denote statistical significance at the p <0.05 level.

EDSSExpanded Disability Status ScaleEQ-VASEQ Visual Analogue ScaleMSmultiple sclerosisQoLquality of LifeSDMTSymbol Digit Modality Test

## Discussion

In this study, we explore the association of several sociodemographic and clinical characteristics, self-reported restricting MS symptoms and perceived support from work, from social and treatment satisfaction with HRQoL among working women with MS. Further, we explore the likelihood of satisfaction with the perceived support in one or more life dimensions (work, social and treatment) among PwMS. Overall, our findings suggest that HRQoL among working women with MS can be individually determined by demographic and disease-related characteristics, self-reported restricting MS symptoms, as well as the perceived support from work, social support or treatment satisfaction. Nevertheless, when all factors are considered, clinical factors such as lower MS severity and fatigue scores, along with reported satisfaction with support from work, emerge as some of the most relevant factors explaining a higher HRQoL among working women with MS. Furthermore, a higher likelihood of reporting satisfaction with perceived support in all three dimensions (work, social or treatment) was associated with lower educational attainment, lower fatigue, having ongoing treatment and higher HRQoL.

Higher HRQoL is generally associated with better overall well-being and functioning. Consequently, individuals with a chronic and disabling condition such as MS, experience reduced HRQoL.^8 9^ Numerous and overlapping factors influence HRQoL in PwMS. In our survey of PwMS, we explored important clinical variables and MS symptoms, especially fatigue, MS severity (focused on physical disability), cognitive processing speed and self-reported MS restrictions, as well as some domains of life affected by MS: support from work, social support, as well as treatment satisfaction. Additionally, factors such as having children at home or civil status were considered, as family composition has shown to have a greater impact on the work capacity of women with MS as compared with men.^5^ Surprisingly, neither of them exerted a significant impact on HRQoL, contradicting earlier research indicating higher HRQoL among single women compared with married/cohabitant, separated or divorced.^23^ However, it is somewhat in line with the observation of no differences in QoL found between mothers with MS and childless women with MS.^24^ Differences in the study population and measures of HRQoL could explain these divergent findings. Moreover, it is important to note that further research is merited given the relatively scarce literature of family composition among women with MS and HRQoL.

Other important sociodemographic factors like education and type of living area exerted a notable impact on HRQoL but lost significance after accounting for all clinical factors and perceived support. For example, consistent with our findings, PwMS living in cities have higher HRQoL, possibly due to better accessibility of essential and clinical resources.^25^ Furthermore, individuals with higher educational levels had higher HRQoL, which could potentially explain greater awareness of healthy behaviours or preventive measures which directly impact their HRQoL, but also fewer physical jobs, greater economic resources and more opportunities for activities that promote health.^26^ Moreover, cognitive difficulties, commonly affected in PwMS,^27^ are also a crucial component of overall well-being, influencing the individuals’ perceived HRQoL. Our findings indicate that higher cognitive processing speed levels are also associated with higher HRQoL, as previously reported.^28^

Additionally, and consistent with other studies from our group, participants who reported having ‘no limiting symptoms’ had a higher HRQoL^4^ and lower MS severity,^21^ compared with those with visible or invisible symptoms. It is highly probable that this finding in our study is due to the fact that most participants were in the early stages of disease progression. While it is known that MS relapses contribute to the accumulation of disability, particularly at the early stages of the disease, the slow progression of disability independent of relapse activity has also shown to become a primary factor behind MS severity.^29 30^ Whether this ‘smouldering’ (asymptomatic) or the relapse activity associated with disease worsening have different impacts on HRQoL is yet to be defined.

Moreover, our findings suggest that, ultimately, lower MS severity (EDSS) and fatigue, satisfaction with perceived support from work as well as certain satisfaction with social support, are among the factors that better predict higher levels of HRQoL. The association between fatigue and/or MS severity with HRQoL has been repeatedly reported, indicating that lower levels of both measures are related to higher HRQoL or vice versa.^2 12 13 31^ Similarly, the impact of support from work on HRQoL has been described among chronic patients, where insufficient support from either supervisors or co-workers was associated with lower HRQoL.^32^ We also observed that having no or unsatisfactory support either from work or social was associated with lower levels of HRQoL, consistent with the literature.^14 15^ Such findings underscore the importance of a supportive work environment and significant others (family, friends or acquaintances) in promoting a higher quality of life for individuals within a wide grade of restrictions.

Importantly, most of the women with MS included in the study had a low to mild level of MS and reported invisible symptoms as the most important MS limitation. The majority also still responded being satisfied or partly satisfied when asked if they receive the support they would like from their closest boss (at least among those that had disclosed). While receiving support from work does not necessarily imply that support is required or sufficient, the notion of perceived support itself still appears to carry significance when coping with MS disease at the workplace.^33^ Altogether, this could be interpreted as perceived support from work being a relevant factor among working women with MS, regardless of the severity of their MS and the ‘evidence’ of the disease’s restrictions. In addition, the impact of the ‘invisible limitations’ of MS, mainly related to fatigue symptoms, cannot be disregarded, as it has previously been shown to affect various aspects of work and private aspects of life (family, leisure activities and contact with friends/acquaintances).^4^ This also accounts for its impact on HRQoL.^6^ Our findings indicated how fatigue, presence of invisible symptoms and cognitive processing speed were associated with HRQoL. Furthermore, the strong and negative association between fatigue and HRQoL prevailed when all other clinical factors were considered. This finding reinforces the idea that fatigue converges with the overall sense of well-being among PwMS,^31^ as a prevalent symptom in PwMS^2^ and work capacity.^6^

Following, we investigated the factors that influence reporting satisfaction in the perceived support of work-life dimensions and treatment. Our findings suggested that satisfaction in all three dimensions was attained in the presence of lower fatigue, lower educational level, ongoing treatment and higher HRQoL. Findings regarding treatment satisfaction are most likely related to the use of DMTs, given that the majority of women in this study had an ongoing DMT prescription during the study period, even at the very early stages of the disease. Hence, early treatment with DMT may potentially uphold a higher HRQoL for PwMS, as it has been shown to delay disability accumulation by years, with the most significant benefit seen in individuals of younger age and relapsing-remitting MS.^29^ However, treatment efficacy and side effects, both unexplored factors in this study, have also shown a significant impact on HRQoL.^34^

Additionally, we unexpectedly found that the likelihood of experiencing satisfactory support in two or three dimensions was higher among those with lower educational levels. In this context, we hypothesise that this association could be linked to lower-skilled and potentially more physically demanding occupations, where the limitations of MS might be more noticeable and result in a greater need for work adjustments and support.^21^ Another possibility is that women with higher educational levels are also more cognizant of the array of resources and support they should ideally receive, thereby being less satisfied with what is currently provided. Initially, these results could reflect a younger and ‘healthier’ MS profile as shown by the unadjusted results. However, after adjusting for age and educational level our findings still sustained, highlighting the relevance of overall well-being and perceived support. While the direction of this association cannot be unravelled, this finding underscores the need for exploring the patient’s work and life circumstances when assessing their HRQoL.

This study has several strengths. It includes a large population-based sample of working-aged women with MS, and their survey responses were subsequently linked with high-quality register data. The web-based survey had a response rate of 71.4% among women, which could be attributed to its convenient mode of administration, enabling participants to complete it at their own pace and in their preferred environment and possessing significant representativeness. However, there are limitations to consider. The findings in this study are linked to the evolving circumstances of the pandemic. Initially, the Swedish National Board of Health and Welfare classified patients with MS as high-risk for COVID-19, but this was later revised to include only those with severe motor impairments affecting respiratory function. These changes may have affected vaccination order for PwMS as well as their exposure to others (at work, healthcare providers, family and friends), due to distancing measures. Further, it is possible that a bias among survey respondents is present, as non-respondents were likely more unwell and may have been unable to cope with answering the survey. Additionally, the large proportion of participants with mild disability (EDSS<3), the age cut-off of 50 years, and the focus on actively working individuals could lead to a representation of a ‘healthier’ MS population, underrepresenting those with more severe illness. Consequently, the findings related to factors influencing perceived HRQoL may be more relevant to individuals with mild MS and may not fully capture the experiences of those with more advanced disease. Nevertheless, it is important to investigate outcomes in early MS that may help shared decision making between patients and physicians (eg, treatment possibilities and alternative approaches, pros and cons of disclosure at the workplace, etc.), which ultimately may lead to enhanced HRQoL. Moreover, the majority of women with MS in this study were currently on medication, while a smaller proportion had no treatment, which was indicative of progressive types of MS. In fewer instances, this included women who were either pregnant or planning to be (data not presented). While the inclusion of ongoing treatment in the multinomial logistic regression could be argued (as considered a dimension of support and thus, implying circularity), we opted to include this factor as the presence of treatment does not necessarily guarantee adherence,^35^ let alone treatment satisfaction. Other limitations to consider involve the cross-sectional design, which reduces our ability to establish causality between all variables and HRQoL, as well as limiting the insights into temporal relationships. Nevertheless, the exploratory nature of this study suggests the potential for further research in HRQoL of PwMS using a more comprehensive approach that goes beyond the clinical perspective.

## Conclusion

HRQoL is a multidimensional concept that can be associated with disease-related characteristics and perceived support from both work and personal life. The interplay between fatigue levels, MS severity and satisfaction with support from the workplace and significant others, can have a considerable impact on the overall well-being of PwMS. However, despite facing severe symptoms, women with MS have shown resilience, maintaining a high level of HRQoL. Implications of these findings could be useful for targeting individuals at risk of a decline in HRQoL: for patients with MS to seek help when fatigue or other invisible MS symptoms appear, and to cultivate a supportive social network and healthy work environment; for healthcare practitioners to appraise beyond the evident MS signs and to inform their patients about the importance of achieving a healthy work-life balance; and for employers to create environments that foster support and well-being among their employees. Furthermore, support for individuals with chronic illnesses could be strengthened by raising community awareness about invisible symptoms and lesser-known aspects of the disease, as well as its effects both in and outside the workplace.

## supplementary material

10.1136/bmjopen-2024-087563 online supplemental file 1

## Data Availability

The data used in the study cannot be made public. Such data can only be made available, after legal review, to researchers who meet the criteria to access such sensitive and confidential data, according to the General Data Protection Regulation, the Swedish Data Protection Act, the Swedish Ethical Review Act and the Swedish Public Access to Information and Secrecy Act. Readers may contact Associate Professor Emilie Friberg (emilie.friberg@ki.se) regarding these data.
