# Supplementary material for: Workplace and social support, treatment satisfaction, and their impact on quality of life in Swedish women with multiple sclerosis: a cross-sectional survey study
Source: BMJ Open. 2024 Dec 20;14(12):e087563. doi: 10.1136/bmjopen-2024-087563 (PMC11667484; doi:10.1136/bmjopen-2024-087563)
Supplement: online supplemental file 1 [file bmjopen-14-12-s001.docx]

**SUPPLEMENTARY MATERIAL**

**
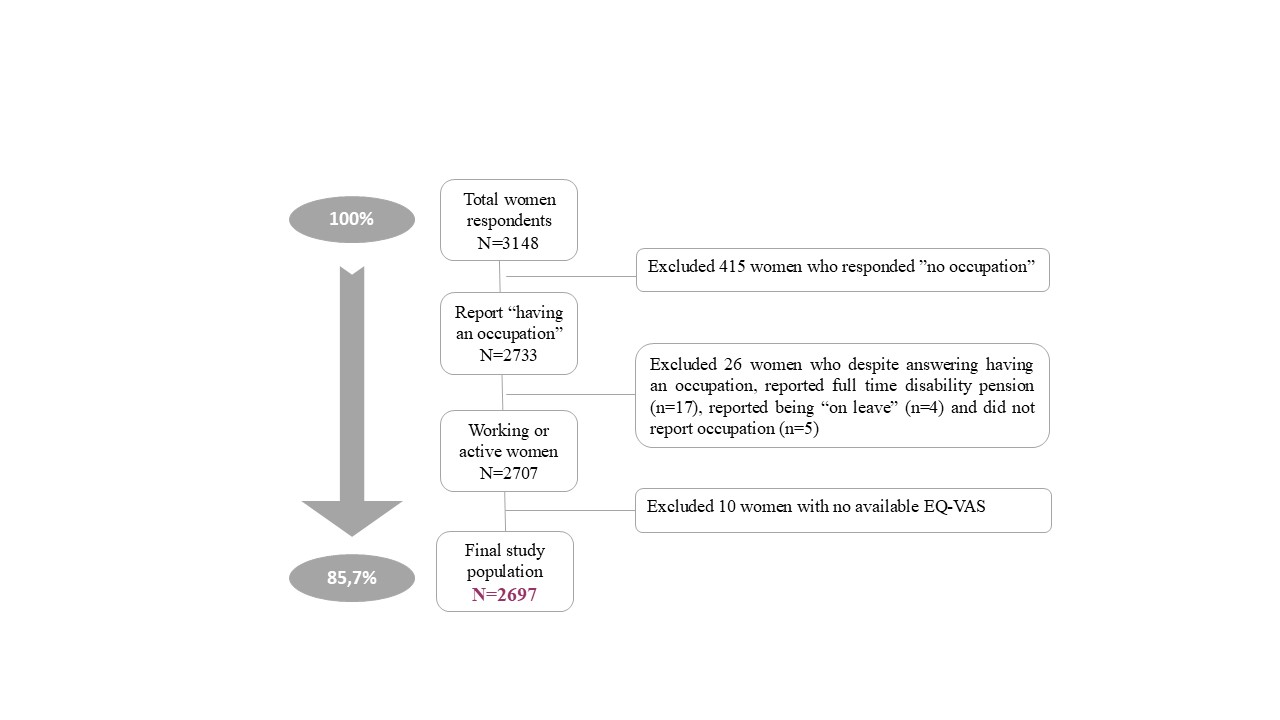
**

**Supplementary Figure 1.** Flow diagram for study participants

**Supplementary Table 1.** Simple Linear regressions for the prediction of Health-related quality of life measure (using EQ-VAS).

| **Predictive variables** | **N** | **R** | **R^2^** | **Unstandardized coefficient** | | **Standardized Coefficient** | **t** | **P-value** |
| --- | --- | --- | --- | --- | --- | --- | --- | --- |
|  |  |  |  | **B** | **SE** | **Beta** |  |  |
| **Age** | 2697 | .028 | .001 | -.065 | .044 | -.028 | -1.466 | .143 |
| **Educational level** (1=University) | 2697 | .069 | .005 | 2.424 | .671 | .069 | 3.612 | **.000** |
| **Country of birth** (1=Sweden) | 2697 | .023 | .001 | 1.191 | 1.009 | .023 | 1.180 | .238 |
| **Children** (1=yes) | 2697 | .010 | .000 | -.340 | .641 | -.010 | -.531 | .596 |
| **Marital/cohabitant status** (1=married/cohabitant) | 2697 | .003 | .000 | -.089 | .629 | -.003 | -.141 | .888 |
| **Type of living area** | 2697 | .027 | .000 |  |  |  |  |  |
| Cities = constant |  |  |  | 75.094 | .469 |  | 160.150 | .**000** |
| Suburbs/towns =1 |  |  |  | -.344 | .691 | -.010 | -.498 | .618 |
| Rural areas =1 |  |  |  | -1.263 | .891 | -.029 | -1.417 | .156 |
| **Type of MS (1=RRMS)** | 2674 | .019 | .019 | 10,519 | 1,466 | .138 | 7,177 | **.000** |
| **Time since diagnosis** | 2524 | .001 | .000 | .061 | .052 | .023 | 1,167 | .243 |
| **Ongoing treatment** | 2697 | .000 | .000 | .094 | 1,086 | .002 | .087 | .931 |
| **Limiting symptoms** | 2392 | .310 | .095 |  |  |  |  |  |
| No symptoms =constant |  |  |  | 87.028 | .854 |  | 101.913 | **.000** |
| Visible symptoms =1 |  |  |  | -13.581 | 1.176 | -.301 | -11.544 | **.000** |
| Invisible symptoms =1 |  |  |  | -14.816 | .932 | -.414 | -15.896 | **.000** |
| **MS Severity (EDSS)** | 2184 | .389 | .151 | -4.334 | .220 | -389 | -19.710 | **.000** |
| **Cognitive processing speed (SDMT)** | 1921 | .154 | .024 | .206 | .030 | .154 | 6.843 | **.000** |
| **Fatigue (Neuro-QoL)** | 2691 | .605 | .366 | -1.034 | .026 | -.605 | -39.386 | **.000** |
| **Support at work*** | 2082 | .036 | .034 |  |  |  |  |  |
| Agree completely =constant |  |  |  | 76.135 | .446 |  | 170.621 | **.000** |
| Agree to some extent =1 |  |  |  | -6.283 | .817 | -.170 | -7.689 | **.000** |
| Do not agree at all =1 |  |  |  | -6.682 | 1.185 | -.124 | -5.641 | **.000** |
| No Answer =1 |  |  |  | .198 | 3.307 | .001 | .060 | .952 |
| **Social support** | 2679 | .069 | .068 |  |  |  |  |  |
| Agree completely = constant |  |  |  | 77.259 | .382 |  | 202.434 | **.000** |
| Agree to some extent =1 |  |  |  | -7.816 | .686 | -.217 | -11.392 | **.000** |
| Do not agree at all =1 |  |  |  | -13.029 | 1.617 | -.152 | -8.060 | **.000** |
| N/A / Have not told =1 |  |  |  | 4.114 | 1.450 | .054 | 2.836 | **.005** |
| **Treatment satisfaction** | 2651 | .054 | 0.53 |  |  |  |  |  |
| True/yes = constant |  |  |  | 76.549 | .342 |  | 223.678 | **.000** |
| Partly =1 |  |  |  | -8.760 | .817 | -.203 | -10.727 | **.000** |
| No =1 |  |  |  | -14.531 | 2.162 | -.127 | -6.721 | **.000** |

**If responded “yes” to disclosure at work.*

*Note*: Predictive variables of more than 2 levels were included as dummy variables (selected level =1) and using one of the levels as reference (constant) in the model)

Abbreviations: EDSS, Expanded disability scale score, SDMT, Symbol digit modality test; HRQoL, Health-related quality of life; EQ-VAS, EQ visual assessment scale

**Supplementary Table 2.** Descriptive characteristics for multinomial logistic regression analysis predicting satisfaction with support at work, social support, and/or treatment

|  | **No support in any dimension** | **Support in 1 dimension** | **Support in 2 dimensions** | **Support in all 3 dimensions** |
| --- | --- | --- | --- | --- |
|  | n=190 | n=692 | n=1009 | n=806 |
|  | n(%) | n(%) | n(%) | n(%) |
| **Age group *** |  |  |  |  |
| 20-29 | 11(5.8) | 48(6.9) | 114(11.3) | 84(10.4) |
| 30-39 | 58(30.5) | 245(35.4) | 315(31.2) | 279(34.6) |
| 40-49 | 108(56.8) | 364(52.6) | 525(52.0) | 387(48.1) |
| 50-51 | 13(6.8) | 35(5.1) | 55(5.5) | 56(6.9) |
| **Educational level** |  |  |  |  |
| Non-University | 48(25.3) | 210(30.3) | 327(32.4) | 277(34.4) |
| University | 142(74.7) | 482(69.7) | 682(67.6) | 529(65.6) |
| **Country of Birth*** |  |  |  |  |
| Sweden | 168(88.4) | 602(87.0 | 897(88.9) | 738(91.6) |
| Other | 22(11.6) | 90(13.08) | 112(11.1) | 68(8.4) |
| **Children <18 at home** |  |  |  |  |
| Yes | 107(56.3) | 442(63.9) | 599(59.4) | 480(59.6) |
| No | 83(43.7) | 250(36.1) | 410(40.6) | 326(40.4) |
| **Married or partnership** |  |  |  |  |
| Married or cohabitant | 84(44.2) | 332(48.0) | 452(44.78) | 385(47.8) |
| Not married/cohabitant, divorced, widowed | 106(55.8) | 360(52.0) | 557(55.2) | 421(52.2) |
| **Type of living area*** |  |  |  |  |
| Cities | 90(47.4) | 336(48.6) | 451(44.7) | 330(40.92) |
| Town/suburbs | 70(36.8) | 233(33.7) | 395(39.1) | 330(40.9) |
| Rural areas | 30(15.8) | 123(17.8) | 163(16.2) | 146(18.1) |
| **MS severity (EDSS) *** | 127(66.8) | 566(81.8) | 829(82.2) | 662(82.1) |
| **Cognitive processing speed (SDMT) *** | 105(55.3) | 494(71.4) | 725(71.9) | 597(74.1) |
| **Fatigue (Neuro-QoL)** | 189(99.5) | 689(99.6) | 1007(99.8) | 806(100) |
| **HRQoL (EQ-VAS)** | 190(100) | 692(100) | 1009(100) | 806(100) |
| **Ongoing treatment *** |  |  |  |  |
| Yes | 155(81.6) | 624(90.2) | 916(90.8) | 754(93.5) |
| No | 35(18.4) | 68(9.8) | 93(9.2) | 52(6.5) |
| **Most limiting symptom *** |  |  |  |  |
| No Symptoms | 8(4.2) | 57(8.2) | 134(13.3) | 127(15.8) |
| Visible symptom | 26(13.7) | 74(10.7) | 150(14.9) | 113(14.0) |
| Invisible symptom | 130(68.4) | 470(67.9) | 622(61.6) | 481(59.7) |
| **Disclosure to boss** |  |  |  |  |
| Yes | 117(61.6) | 453(65.5) | 709(70.3) | 806(100) |
| No/missing | 73(38.4) | 239(34.5) | 300(29.7) | 0(0) |

*Abbreviations*: EDSS, Expanded disability scale score, SDMT, Symbol digit modality test; HRQoL, Health-related quality of life; EQ-VAS, EQ visual assessment scale

***** Different proportions among number of supports were compared using Chi-square test with a significance level of 0.05. Tests are adjusted for all pairwise comparisons using the Benjamini-Hochberg correction.

*Note*: Support in a dimension is considered when responding “completely agree” to survey questions related to receiving support from work and/or family and if responding “yes” to question related to treatment satisfaction.

**Supplementary Table 3** Complementary multinomial logistic regression analysis for all participants who had disclosed their Multiple sclerosis (MS) condition at work

|  | **Adjusted OR [95% CI]** | | |
| --- | --- | --- | --- |
|  |  | | |
|  | **Support in 1 dimension** | **Support in 2 dimensions** | **Support in all 3 dimensions** |
| **Age group** |  |  |  |
| 20-29 | 1.587 [.527 – 4.780] | 2.619 [.896 – 7.659] | 2.492 [.839 – 7.404] |
| 30-39 | 1.311 [.775 – 2.217] | 1.176 [.699 – 1.979] | 1.343 [.789 – 2.288] |
| 50+ | 1.190 [.416 – 3.403] | 1.234 [.441 – 3.455] | 1.448 [.501 – 4.186] |
| **Educational level** |  |  |  |
| Non-University | 1.392 [.803 – 2.414] | **1.724 [1.003 – 2.965]** | **2.062 [1.185 – 3.588]** |
| **MS severity (EDSS)** | .935 [.789 – 1.106] | 1.045 [.886 – 1.231] | 1.026 [.864 – 1.219] |
| **Cognitive processing speed (SDMT)** | .999 [.979 – 1.018] | 1.012 [.993 – 1.031] | 1.013 [.993 – 1.033] |
| **Fatigue (Neuro-QoL)** | **.961 [.928 – .996]** | **.930 [.898 – .963]** | **.919 [.886 – .952]** |
| **HRQoL (EQ-VAS)** | 1.007 [.990 – 1.024] | 1.015 [.999 – 1.032] | **1.033 [1.015 – 1.052]** |
| **Ongoing treatment** |  |  |  |
| No | **.368 [.173 - .785]** | **.356 [.170 - .743]** | **.201 [.088 - .461]** |
| **Most limiting symptom** | |  |  |
| Visible symptom | .506 [.129 – 1.996] | .515 [.135 – 1.968] | .513 [.133 – 1.981] |
| Invisible symptom | .786 [.219 – 2.822] | .710 [.203 – 2.488] | .769 [.218 – 2.709] |

*Abbreviations*: OR, Odd ratios; CI, confidence intervals; EDSS, Expanded disability scale score, SDMT, Symbol digit modality test; HRQoL, Health-related quality of life; EQ-VAS, EQ visual assessment scale

References (predictive variables): Age (40-49 years), Educational level (university), ongoing treatment (yes) and most limiting symptom (no symptom).

Reference (dependent variable): No Support

Adjusted model samples size is n=1330.

**Annex**

# MS Survey – extraction of the web-based survey translated to English

**What is your current occupation?** (Enter all that apply, multiple answers are possible)

Employed

Own company/own business

Old age pensioner

Student/work experience placement

On parental leave

On sick leave

On disability pension, part-time

On disability pension, full-time

On leave of absence

Job seeker / unemployed

Other, what: _____

**What type of employment contract do you have?** (If you have several employments, tick your main employment)

Permanent employment

Fixed-term employment (temporary, probationary, seasonal)

Hourly employment

Wage subsidy employment

Other: ____

**Has your employment been affected by the ongoing COVID-19 pandemic?** (multiple answers are possible)

No

Yes, I am furloughed ___ % of full-time

Yes, I have been furloughed ___ % of full-time

Yes, I am unemployed (with compensation)

Yes, I am unemployed (without compensation)

Yes, I am employed and have had less to do

Yes, I am employed and have had more to do

Yes, I have started to study or pursue further education

Other: _____

**How has the ongoing COVID-19 pandemic affected you in respect of being self-employed?** (Answers: No / partly / yes)

My business has gone better than before

I have mostly been able to work as usual

I have digitalized my business activities

I have undertaken other types of assignment

My business has gone down but I have managed to keep it going

The business is dormant

I have dissolved my business

My business has gone into bankruptcy

**We would like to know how good or bad your health is TODAY. The scale is numbered from 0 to 100. 100 is the best health you can imagine. 0 is the worst health you can imagine.**

Fill in a number between 0 and 100: _____

**Do you, or have you had COVID?**

Yes, and it is confirmed with a test

Yes, I think so but have not had it confirmed with a test

No

I do not know

**How do you feel now?**

I am sick / less than two months since I fell ill

Fully recovered

Remaining symptoms

**Which remaining symptoms do you have?**

Text: _____

**Has the COVID-19 pandemic permanently affected your occupation in any of the following ways?** (Answers: Yes / Yes, to some extent / No / Not applicable)

Received / undertaken other tasks

Decreased working hours

Changed job

Became self-employed

Became employed

Quit your job

Started / applied for an education

**In general, how has your health been affected by the ongoing COVID-19 pandemic?**

Very much improved

Slightly improved

About the same

Slightly worsened

Very much worsened

**What is your current occupation?** Write in as much detail as possible about your main occupation (e.g. assistant nurse at a nursing home, journalist at a daily newspaper, nanny at a preschool, HR consultant at a municipal personnel department)

I am not in paid work

OR Text: _____

**Have you told the following people at your workplace about your MS-diagnosis?** multiple answers are possible. If you have multiple employments, answer the statements for your main employment

*(Answers: Yes; No, but I will; No; Not applicable/do not have)*

To my boss

To most / all co-workers

To certain co-workers

To employees

To the HR-department

To occupational healthcare

To customers / patients / clients / students

**The following statements concern what support you have received from your work and what support you wished you had received for your MS** (Answers: Do not agree at all; Agree to some extent; Agree completely)

“My closest boss supports me by…”

… showing compassion and understanding

… giving advice to manage work life

… considering my disease when planning work tasks

I receive the support I would like from my closest boss

**Do you have any adjustments/support that helps you with your work?**

Yes

Yes, but not enough

No, but I do need

No, not needed

**If you consider the adjustments/support that facilitate, or could facilitate paid work, which are the three most important?**

Text 1: ____

Text 2: ____

Text 3: ____

**To what extent do you experience that your MS limits you in the following situations?**

(Answers 1= not at all - 7=to a very high extent; 8=Not applicable)

Work situation

Family situation

Leisure activities

Contacts with friends and acquaintances

**Which MS symptom do you experience as the most limiting?**

Text: ____

**What treatment/s do you currently have for your MS?**

Text: ____

**Are you satisfied with your treatment?**

Yes

Partly

No

**Please motivate your answer**

Text: ____

**The following statements concern the support you have received from your friends/family in relation to your MS** (Answers: Do not agree at all; Agree to some extent; Agree completely; Not applicable; Have not told)

My friends/family give me support by …

Showing compassion and understanding

Giving advice to manage work life

I receive the support I wish from my friends and family
