# Supplementary material for: Workplace and social support, treatment satisfaction, and their impact on quality of life in Swedish women with multiple sclerosis: a cross-sectional survey study
Source: BMJ Open. 2024 Dec 20;14(12):e087563. doi: 10.1136/bmjopen-2024-087563 (PMC11667484; doi:10.1136/bmjopen-2024-087563)
Supplement: Supplementary file 2 [file bmjopen-2024-087563.reviewer_comments.pdf]

PEER REVIEW HISTORY

BMJ Open publishes all reviews undertaken for accepted manuscripts. Reviewers are asked to complete a checklist review form and are provided with free text boxes to elaborate on their assessment. These free text comments are reproduced below.

ARTICLE DETAILS

Title (Provisional)

Workplace support, social support, and treatment satisfaction and their impacts on quality of life in Swedish women with multiple sclerosis: a cross-sectional survey study

Authors

Machado, Alejandra; Wredendal, Elin; Fink, Katharina; Friberg, Emilie

---

VERSION 1 - REVIEW

**Reviewer**	**1**
**Name**	**O'Shea, Dede**
**Affiliation**	**UMass Chan**
**Date**	**24-May-2024**
**COI**	**None**

Methods

Was there confirmation that the continuous variables such as fatigue, cognitive processing speed were normally distributed?

Results

A few particular correlations are reported in table notes. It would be helpful to have a full correlation table for the continuous predictor variables and outcome measures to understand the linear relationships.

Discussion

Participants enrolled in the study had largely mild EDSS disability. There is discussion that lower MS severity predicted high HrQOL. What effect would the study sample, and its limitation, be considered to have on this finding?

**Reviewer**	**2**
**Name**	**Mustač, Filip**

Affiliation **University Clinical Hospital Center Zagreb, Department of Psychiatry and Psychological Medicine**

Date **19-Jul-2024**

COI **None to declare.**

Summary:

This cross-sectional designed study aims to assess health-related quality of life (HRQoL) and connection with support from friends, family and work in the population of women with multiple sclerosis who are employed. The study is based on the population of 2967 working women who met all eligible criteria. Firstly, they investigated Nationwide Swedish register/Swedish MS Registry (SMSreg) according to those criteria and then people with multiple sclerosis were invited to take a part in an online survey in the period from May to September 2021. They used registers variables as well as survey measures which they have categorized into three categories: No symptom, visible symptoms, invisible symptoms. Their results showed that HRQoL is connected to MS severity, support from work and fatigue in a way that higher levels of HRQoL were connected to higher processing speed, educational attainment, and lower MS severity and fatigue. On the other side, working women with lower HRQoL reported none or unsatisfactory support from people around them, as well as they reported more visible or invisible symptoms. They concluded that satisfaction with the support from work can be of important significance when we talk about working women with MS.

Importance in the field:

This article is presenting an interesting study which raises the question of the importance of support of other people in the daily life of people with a chronic and potentially very disabling disease such is multiple sclerosis. Since we still spend a good part of our time dedicated to our work no matter how much we try to be professional and independent, relationships with people at work leave a significant impression on our daily lives. Therefore, studying relationships with other people who are important to us and who provide us with support in dealing with the disease makes a lot of sense. This paper contributes precisely by studying the support of other people in women with multiple sclerosis.

Major comments:

• The abstract is generally well written. The only thing that could possibly be useful is to highlight in the abstract that the research was conducted as online research.

• Since the online research was conducted from May to September 2021, it should also be pointed out that the support from the colleagues, family and friends worked then in the era of much changed circumstances, such as they were during the COVID-19 pandemic. Could it be related to the research results? That should certainly be commented on and supported by the literature in the discussion and limitations of the research.

• Furthermore, people with chronic diseases were especially emphasized during the pandemic as those who are at risk and who should be extremely protected and isolated. Any information in the literature about how people with multiple sclerosis responded to vaccination and how the most of them functioned at that time would certainly be useful to comment on in the discussion.

• Precisely because of the complexity of the problem and support for people with demanding diagnoses, perhaps the introduction could be supplemented with a short review of the literature that explains in more detail what would be the reasons for studying this topic through the prism of support from people at work. Why is the acceptance and support of people at work the very factor that is important to investigate, and not, for example, the support of the social system in general or the existence of psychological support groups for people with multiple sclerosis. It can also be addressed in more detail in the very introduction of the paper.

• Could you describe in the discussion or in another part of the manuscript why the results of the survey for men are not shown since 1264 solved the questionnaire according to the same methodology. I agree that it is probably out of scope of this paper because in this paper you decided to show the results on the population of working women, but since it is in the Methods shown how many men answered the questionnaire, it would probably be good to comment on it in some part of manuscript.

• The results are clearly presented and written.

• The discussion is mostly clearly written and opens questions arising from the study itself. One of the suggestions is the very nature of online research. Could you comment on the screening/sampling of the participants. Since the research is online, it could possibly be related to the fact that those people who have a more severe or disabling form of MS may not have answered the questionnaire in the survey. This could possibly be a limitation of the study as well.

Minor comments:

• It might be interesting to comment in the discussion on what other forms of support are important for people with MS and how they could be improved in such a way that we as a community do as much as possible for people with MS.

Overall, the paper presents a new insight into the importance of quality of life in people with multiple sclerosis.

**Reviewer**	**3**
**Name**	**Safar, Laura**
**Affiliation**	**Harvard Medical School, Psychiatry**

Date **22-Jul-2024**

COI **None.**

Thank you for the opportunity to review this study, and thank you to the Authors for your work in this important topic. Below are my comments for your consideration.

BMJ Checklist Question #2: Is the abstract accurate, balanced and complete?

I answered "no" mostly for two reasons: a) It seems unclear to what degree the authors found that demographic factors have an impact on HRQoL. The only demo factor they include in the abstract's results section is educational attainment. It is unclear to me if educational attainment continues to be relevant as factor associated with HRQoL once clinical factors (e.g. MS severity, etc) are controlled for.

b) As reminder to edit the abstract if changes take place as result of the review process.

BMJ Checklist Question #4: I defer to a reviewer with higher expertise in research methodology to answer this question.

BMJ Checklist Question #7: I defer to a reviewer with higher expertise in research methodology to answer this question.

BMJ Checklist Questions #9,10,11,12: Please refer to my questions and comments in the attachment.

Manuscript: Support from work, family and friends, and treatment satisfaction in health-related quality of life among working women with MS.

Reviewer: Laura T Safar, MD

Note: The comments and questions below follow the various sections of the manuscript. They skip the Abstract- however edits to the Abstract may be required as result of edits in the main body of the manuscript.

Data sources and study population. Page 5, lines 48-60.

Please explain the decision to use the cutoff of 50 years old. Extending the study to an older age (i.e. 60) would have possibly captured a larger proportion of women with more severe MS and possibly, with different findings in the variables you are studying.

Similarly: I understand that this is a study of working women with MS, and for this reason non-working women were excluded (about 14% of the women-respondents). I am curious what subset of this 14% are not working due to disability from MS, or due to lack of appropriate support at work, for instance. I think this could have contributed to the authors capturing a sample with less severe MS in their study.

86% of the women who responded the survey are working. How does this compare to Swedish women ages 20-50?

The online survey took place between May and September 2021- this was still a time highly impacted by the pandemic, for instance with larger proportion of people working from home. Please comment somewhere in the manuscript (discussion?) if/ how the timing of the survey may have affected the results. For instance, is working from home perhaps less demanding on pwMS's decreased energy, and therefore their relative need for adjustments and support from employers less marked than when working at the workplace?

Register Variables. Page 6, lines 7-28.

-Consider discussing the limitations of EDSS, for instance in regards to non-visible MS symptoms such as fatigue, depression, cognitive dysfunction. This may be relevant regarding a high percentage of your sample with mild MS severity however a large percentage of them report being affected by non-visible symptoms.

Survey measures. Page 6, lines 59-60.

"Are you satisfied with your treatment?" is a very open-ended question. Somewhere else in the manuscript (page 10, lines 49-50) the authors mention the hypothesis that subjects possibly answered this thinking of their use of DMTs. I do not see data here to support this hypothesis. Treatment satisfaction can be influenced by multiple factors: Satisfaction with their clinicians; satisfaction with the support staff; with access to timely appointments; cost of care; effectiveness of medication; side effects; their perceived severity of their MS or their perceived disability can impact negative perception about treatment. It can also be affected by depression (lifetime prevalence in MS 25-50%), which can result in negative perceptions about several variables including this one.

Results:

Page 7 Lines 50-51: While 53.5% of respondents were not married/not in partnership, 60.4% had children living at home. Are these percentages comparable to Sweden's general female population? Please disregard if they are comparable- I am curious about a higher percentage of non-partnered women due to their MS.

Lines 53-54: Cut off of 50y/o and excluding non-working women could have contributed to this sample presenting high percentage of RRMS with low EDSS.

Page 8 line 14: There seems to be something missing in that sentence. For instance …had a positive association with HRQoL ***and*** were associated with higher HRQoL.(?)

Broadly speaking, it seems that (as expected, and previously reported): Worse clinical profile is associated with worse HRQoL; milder clinical profile is associated with better HRQoL. Please list more clearly / separate clearly your findings regarding the other variables (satisfaction with each type of support; satisfaction with treatment) and their association with HRQoL once MS severity/ illness factors are controlled for. Please see the first paragraph below, I feel that further clarification

in the results section may be also needed regarding the impact of demographic factors once clinical factors are controlled for.

General, for Results: The “Invisible symptoms” include many problems highly prevalent in MS. In addition to fatigue and cognitive difficulties, pain and depression (and anxiety). I am curious if you have access to pain scores for this sample, or depression/ anxiety (emotional distress) scores to disentangle this variable.

Discussion.

Page 9, lines 10-11: “our findings suggest that HRQoL …can be determined by demographic. ..characteristics”. Please clarify. The way this statement reads, it places demographic characteristics at the same level as disease-related characteristics. However in the Results: it seems that demographic characteristics become less important once adjusted for other factors (Page 8, lines 13 and on: “However, when all key factors are considered…”. This is also mentioned in Discussion, page 9 line 44. Please clarify. I think a more accurate way to discuss your findings may be that demographic factors at large were NOT associated w HRQoL once other variables were controlled (except for one or two exceptions).

Page 9, lines 18-19. I am not sure I would clump together “treatment” with the other two types of perceived supports. As discussed previously satisfaction with treatment may involve a number of factors including biological effectiveness.

Page 9 line 26: women handle MS impact more effectively than men: this is interesting, but it is not studied (duplicated or not) here.

Page 9 lines 27-28: I would distinguish clinical factors and symptoms (MS severity, fatigue etc) from “domains of life” such as support; would not use the same collective noun for all. This is semantics, does not affect the findings.

Page 9 lines 28-29: “MS severity (focused on physical disability)”: Consider commenting on the limitation that because you used a cutoff of age 50 and because you excluded female respondents who are not working, you may have captures a less disabled sample therefore this aspect of MS was not as significantly included in this study.

Page 10 lines 9-11: As discussed before, I am curious how clear the influence of satisfaction with perceived support is, after controlling for disease severity.

Page 10 lines 26-27: Please briefly comment on the legislation in Sweden regarding employers’ obligation to provide support once people disclose an illness. Related to this, please also comment on percentage of people in Sweden who disclose their illnesses at work.

Page 10 lines 33-34: The invisible limitations of MS include other problems that are also (as fatigue is) very prevalent in MS- including pain, depression, and cognitive dysfunction. Please consider editing this to include these other phenomena.

Page 10 line 49: as I mentioned above, "treatment satisfaction" can include a large number of aspects. Not sure how we know here that respondents were thinking of DMTs when they answered; we did not ask them.

VERSION 1 - AUTHOR RESPONSE

Reviewer: 1
Dr. Dede O'Shea, UMass Chan
Comments to the Author:

We would like to thank the reviewer for taking the time to review this manuscript and providing valuable comments.

Methods
Was there confirmation that the continuous variables such as fatigue, cognitive processing speed were normally distributed?

Author's response: Yes, as shown in Table 1 and histograms below, the skewness of both variables, as well as kurtosis of the fatigue score are excellent (between -1 and +1), while the kurtosis of the cognitive processing speed score (SDMT) is under acceptable values. A statement of normal distribution confirmation has been added to the revised manuscript (page 6, statistical analysis, paragraph 1).

Table 1. Descriptive values and normal distribution parameters for Fatigue and SDMT

Descriptive Statistics

	N	Minimum	Maximum	Mean	Std. Deviation	Skewness		Kurtosis	
	Statistic	Statistic	Statistic	Statistic	Statistic	Statistic	Std. Error	Statistic	Std. Error
fatigue_T_score	2691	29.5	74.1	50.406	9.5102	-.110	.047	-.283	.094
SDMT score past 3 years	1921	11	110	58.12	12.118	.453	.056	1.771	.112

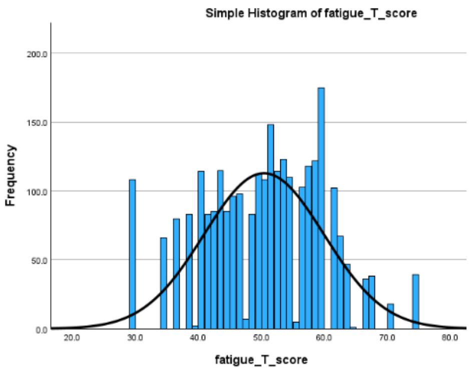

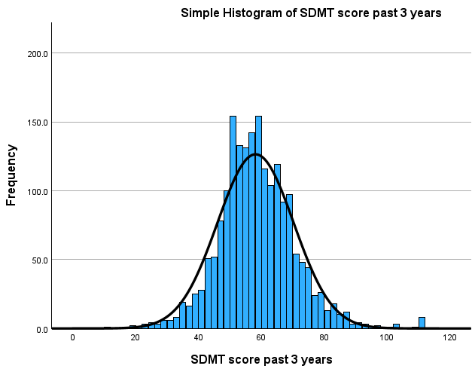

Results

A few particular correlations are reported in table notes. It would be helpful to have a full correlation table for the continuous predictor variables and outcome measures to understand the linear relationships.

Author's response: We thank the reviewer for this suggestion. All predictor variables are significant correlated (p<.001) to the outcome (EQ-VAS). The Fatigue score showed a strong negative relationship to EQ-VAS (r=-.605), while the effect sizes from EDSS (r=-.389) and SDMT (r=.154) with EQ-VAS differed, respectively, from moderate to weak, as shown in the Table 2. Moreover, and as expected, these clinical predictor variables are also significantly correlated across them. To note, similar values resulted in the correlation matrix of the regression model, where multicollinearity was assessed and discarded: all variables in the correlation matrix were under 0.80, variance inflation factor (VIF) values were all below the standard used threshold of 4 and above 0.25 for Tolerance indicator.
An indication of assumptions for multiple linear regression analysis have been added to the methods section of the revised manuscript (page 6, statistical analysis section, paragraph 2).

Table 2. Correlations between continuous predictive variables and outcome variable EQ-VAS

Correlations

		fatigue_T_score	Most recent EDSS measurement	SDMT score past 3 years	EQ_VAS
fatigue_T_score	Pearson Correlation	1	**.288****	**-.134****	**-.605****
	Sig. (2-tailed)		<.001	<.001	<.001
	N	2691	2179	1917	2691
Most recent EDSS measurement	Pearson Correlation	.288**	1	**-.199****	**-.389****
	Sig. (2-tailed)	<.001		<.001	<.001
	N	2179	2184	1913	2184
SDMT score past 3 years	Pearson Correlation	-.134**	-.199**	1	**.154****
	Sig. (2-tailed)	<.001	<.001		<.001
	N	1917	1913	1921	1921
EQ_VAS	Pearson Correlation	-.605**	-.389**	.154**	1
	Sig. (2-tailed)	<.001	<.001	<.001	
	N	2691	2184	1921	2697

**. Correlation is significant at the 0.01 level (2-tailed).

Discussion

Participants enrolled in the study had largely mild EDSS disability. There is discussion that lower MS severity predicted high HrQOL. What effect would the study sample, and its limitation, be considered to have on this finding?

Author's response: We agree with the reviewer that a milder level of disability (assessed by the EDSS scale) among the sample is a limitation to our study, which has been included in the revised manuscript (page 10, paragraph 2).

Unfortunately, we could not capture the association of HRQoL when exploring correlations from the moderate (n=289, r=-.033, p=.582) and severe EDSS (n=47, r=-.108, p=.472) levels included in our study. We presume this is due to the small representation of participants with EDSS≥3. However, we believe that people with Multiple sclerosis (PwMS) with these higher levels of disability would report lower HRQoL. This presumption is based on the results from McKay et al. (2020),[1] using a bigger sample of the same nationwide Swedish MS register (SMSreg) as used in our study. McKay and colleagues explored the determinants of quality of

life (using EQ-VAS measure) among PwMS over time. Their results indicated that among PwMS with adult onset, higher levels of disability were consistently associated with lower HRQoL. These results were found to be in line with previous findings.[2, 3]

Reviewer: 2
Dr. Filip Mustač, University Clinical Hospital Center Zagreb
Comments to the Author:

Summary:
This cross-sectional designed study aims to assess health-related quality of life (HRQoL) and connection with support from friends, family and work in the population of women with multiple sclerosis who are employed. The study is based on the population of 2967 working women who met all eligible criteria. Firstly, they investigated Nationwide Swedish register/Swedish MS Registry (SMSreg) according to those criteria and then people with multiple sclerosis were invited to take a part in an online survey in the period from May to September 2021. They used registers variables as well as survey measures which they have categorized into three categories: No symptom, visible symptoms, invisible symptoms. Their results showed that HRQoL is connected to MS severity, support from work and fatigue in a way that higher levels of HRQoL were connected to higher processing speed, educational attainment, and lower MS severity and fatigue. On the other side, working women with lower HRQoL reported none or unsatisfactory support from people around them, as well as they reported more visible or invisible symptoms. They concluded that satisfaction with the support from work can be of important significance when we talk about working women with MS.

Importance in the field:
This article is presenting an interesting study which raises the question of the importance of support of other people in the daily life of people with a chronic and potentially very disabling disease such is multiple sclerosis. Since we still spend a good part of our time dedicated to our work no matter how much we try to be professional and independent, relationships with people at work leave a significant impression on our daily lives. Therefore, studying relationships with other people who are important to us and who provide us with support in dealing with the disease makes a lot of sense. This paper contributes precisely by studying the support of other people in women with multiple sclerosis.

We would like to thank the reviewer for his feedback and for taking the time to review this manuscript.

Major comments:

• The abstract is generally well written. The only thing that could possibly be useful is to highlight in the abstract that the research was conducted as online research.

Author's response: We thank the reviewer for this suggestion which has been added to the 'Design' section of the abstract.

• Since the online research was conducted from May to September 2021, it should also be pointed out that the support from the colleagues, family and friends worked then in the era of much changed circumstances, such as they were during the COVID-19 pandemic. Could it be related to the research results? That should certainly be commented on and supported by the literature in the discussion and limitations of the research.

Author's response: Indeed, the changing circumstances of COVID-19 likely influenced the broader social network around the participant. Our recent publication, based on the same survey data, highlighted how social networks helped individuals with MS navigate both their condition and the added stress of the pandemic.[4] This study, which examined the working life

of PwMS during COVID-19, also highlighted the broader impact on personal wellbeing, social and family relationships, and work environments, including improved understanding and support from work colleagues. However, participants also expressed concerns about contracting SARS-CoV-2, adopting safer behaviours like physical distancing and working from home.[4, 5] This behaviour, together with the pandemic restrictions, may have reduced the support network for individuals with chronic disease (e.g., healthcare providers, family, and friends), a situation that was not uncommon during the pandemic.[6] These factors may have influenced our findings, as support from significant others, family, and friends is linked to better HRQoL in people with MS (PwMS).[7] In this regard, our results showed a stronger association between support from work and HRQoL compared to support from family and friends, which was present but to a lesser degree. Therefore, the reviewer's hypothesis on this matter is probably correct: the changing COVID-19 circumstances also affecting family and friends of PwMS might have affected our findings (e.g., reduced contact with the PwMS to avoid risk of infection - either by own initiative or due to the patient's will). These implications are now included in the discussion section of the revised manuscript (page 10, lines, 265-271).

• Furthermore, people with chronic diseases were especially emphasized during the pandemic as those who are at risk and who should be extremely protected and isolated. Any information in the literature about how people with multiple sclerosis responded to vaccination and how the most of them functioned at that time would certainly be useful to comment on in the discussion.

Author's response: We thank the reviewer for this remark. Vaccination against COVID-19 started in December 2020, after EU approval for Pfizer–BioNTech vaccine. Risk groups and older age groups (70+ years) were first invited, extending this invitation to individuals aged 65 years and older in beginning of March 2021. By April 2021, 23% of individuals over 65 years and 8% of the Swedish population as a whole were fully vaccinated.[8] Unfortunately, we do not have information whether the participants with MS in our study were vaccinated or not. However, considering the age ranges of our participants, we infer most of the them had not received a first dose by the time the survey was completed (May 2021) but we have no data to confirm this.

In terms of how most of them were managing at that time, the Swedish National Board of Health and Welfare initially classified MS patients as a high-risk group for COVID-19, prompting requests for work adjustments and remote work options.[9] However, the classification was later revised to include only those with severe MS-related motor impairments, particularly affecting respiratory function. [10] In spring 2020, the government introduced preventive sickness compensation for risk groups, followed by temporary sickness compensation for those unable to work from home. This support extended to relatives needing to avoid work to protect high-risk family members, with the qualifying period lasting until April 2021.[11]

This information is now included in the discussion section of our revised manuscript (page 10, paragraph 2).

• Precisely because of the complexity of the problem and support for people with demanding diagnoses, perhaps the introduction could be supplemented with a short review of the literature that explains in more detail what would be the reasons for studying this topic through the prism of support from people at work. Why is the acceptance and support of people at work the very factor that is important to investigate, and not, for example, the support of the social system in general or the existence of psychological support groups for people with multiple sclerosis. It can also be addressed in more detail in the very introduction of the paper.

Author's response: We thank the reviewer for this suggestion. Since MS is often diagnosed in young adults during the midst of their careers, support from work becomes a crucial factor. While some may be temporarily on sick leave or working part-time, most can continue working for extended periods. Besides, employment not only enhances their quality of life but also serves as an indicator of overall functioning.[12] Hence our interest in exploring the prism of support from work. While examining support from the social system or psychological support groups would have been a valuable addition, we believe these influences may have been more volatile due to the uncertainty and constantly shifting circumstances of the pandemic. A short comment on this respect has been included in the introduction section of our revised manuscript (page 4, Introduction, paragraph 2).

• Could you describe in the discussion or in another part of the manuscript why the results of the survey for men are not shown since 1264 solved the questionnaire according to the same methodology. I agree that it is probably out of scope of this paper because in this paper you decided to show the results on the population of working women, but since it is in the Methods shown how many men answered the questionnaire, it would probably be good to comment on it in some part of manuscript.

Author's response: We thank the reviewer this this suggestion. The main reason for this was the higher response rate on the Survey among women (n=3148; 71,4%) compared to men (n=1264, 28.6%). We acknowledge this might be confusing for the reader. Thus, for clarity, we will remove the men criteria in the methods section and flow chart figure in the Supplementary file.

• The results are clearly presented and written.

Author's response: we thank the reviewer for the feedback.

• The discussion is mostly clearly written and opens questions arising from the study itself. One of the suggestions is the very nature of online research. Could you comment on the screening/sampling of the participants. Since the research is online, it could possibly be related to the fact that those people who have a more severe or disabling form of MS may not have answered the questionnaire in the survey. This could possibly be a limitation of the study as well.

Authors' response: Overall, the non-responders are characterized by higher proportions of men, younger patients, born outside Sweden and with none or low income. Further, patients with 5-9 years since diagnosis of MS responded in lesser extent while those with longer disease duration (and probably with more progression) had a higher coverage rate. Unfortunately, we can not determine more clinical characteristics of the non-responders as the survey was administered by Statistics Sweden, an external Swedish authority that linked the sociodemographic data of the respondents to the clinical register, before the anonymization and delivery of the dataset. We agree with the reviewer that the mild levels of disability of our sample may present a limitation. We have addressed this limitation in our study (page 10, paragraph 2).

Minor comments:

• It might be interesting to comment in the discussion on what other forms of support are

important for people with MS and how they could be improved in such a way that we as a community do as much as possible for people with MS.

Author's response: We have included this suggestion in the conclusion section of the revised manuscript (page 11).

Overall, the paper presents a new insight into the importance of quality of life in people with multiple sclerosis.

Author's response: We sincerely appreciate the reviewer's thoughtful feedback.

Reviewer: 3 [PLEASE SEE ATTACHED DOCUMENT FOR FULL/ADDITIONAL COMMENTS FROM REVIEWER 3]
Dr. Laura Safar, Harvard Medical School
Comments to the Author:
Thank you for the opportunity to review this study, and thank you to the Authors for your work in this important topic. Below are my comments for your consideration.

We appreciate the reviewer's insightful comments and suggestions. To avoid repetition, we will address these comments more in-depth following the reviewer's observations included in the attachment file. Please see below.

BMJ Checklist Question #2: Is the abstract accurate, balanced and complete?
I answered "no" mostly for two reasons: a) It seems unclear to what degree the authors found that demographic factors have an impact on HRQoL. The only demo factor they include in the abstract's results section is educational attainment. It is unclear to me if educational attainment continues to be relevant as factor associated with HRQoL once clinical factors (e.g. MS severity, etc) are controlled for.

b) As reminder to edit the abstract if changes take place as result of the review process.

BMJ Checklist Question #4: I defer to a reviewer with higher expertise in research methodology to answer this question.
BMJ Checklist Question #7: I defer to a reviewer with higher expertise in research methodology to answer this question.
BMJ Checklist Questions #9,10,11,12: Please refer to my questions and comments in the attachment.

----------ADDITIONAL COMMENTS FROM REVIEWER 3 FROM ATTACHED DOCUMENT --------------------

Manuscript: Support from work, family and friends, and treatment satisfaction in health-related quality of life among working women with MS.
Reviewer: Laura T Safar, MD

Note: The comments and questions below follow the various sections of the manuscript. They skip the Abstract- however edits to the Abstract may be required as result of edits in the main body of the manuscript.

Author's response: We appreciate the reviewer for highlighting this issue, as we may not have adequately conveyed our conclusions. There are two different types of results involving the demographic variables: one is regarding the influence on HRQoL and the other, on the different number of support dimensions (work, family and friends, and treatment satisfaction). On the former, higher educational attainment and living in cities were associated with higher HRQoL when performing simple linear regressions (Table 1, in Supplementary file). However, when introducing these variables in the multiple logistic regression (Table 3 in the manuscript), the effect of these variables disappears. This finding is described in the discussion section of the manuscript "Other important sociodemographic factors like education and type of living area exerted a notable impact on HRQoL but lost significance after accounting for all clinical factors and perceived support" (page 8, lines 194-195).

On the other hand, the impact of demographic variables in the number of support dimensions (unadjusted multinomial logistic regression- Table 4 in manuscript), suggested that younger age and non-university education had a stronger likelihood with support on two and all three dimensions (work, social, and treatment satisfaction). However, when studying these variables with the rest of clinical and symptomatic variables (adjusted models), the association of age with HRQoL became insignificant, probably captured by the progressive effect of the disease (EDSS and ongoing treatment), while the effect of education remained. This negative association with HRQoL is discussed in the revised manuscript (pages 10, paragraph 2).

We have revised the abstract accordingly to minimize the emphasis on the demographic factors.

Data sources and study population. Page 5, lines 48-60.
Please explain the decision to use the cutoff of 50 years old. Extending the study to an older age (i.e. 60) would have possibly captured a larger proportion of women with more severe MS and possibly, with different findings in the variables you are studying.

Author's response: We thank the reviewer for this remark. The general aim of the survey study was to gain insights in the life-situations of people with MS around the time when they made decisions about work, family, career etc. While we acknowledge that our findings would differ if capturing women over 50 years and, possibly with more severe MS, we chose to focus on a sample between 20-50 years to explore working life and life balance.

Similarly: I understand that this is a study of working women with MS, and for this reason non-working women were excluded (about 14% of the women-respondents). I am curious what subset of this 14% are not working due to disability from MS, or due to lack of appropriate support at work, for instance. I think this could have contributed to the authors capturing a sample with less severe MS in their study. 86% of the women who responded the survey are working. How does this compare to Swedish women ages 20-50?

Author's response: This percentage of non-working women with MS had higher levels of disability and disease progression, however, we do not know the exact reason for why they aren't working. Unfortunately, the survey design caused participants to skip all work-related questions (e.g., support, work adaptations) if they selected "not in work." Thus, we have no information if they are not working due to severe symptoms or lack of appropriate support at work, for example.

Regarding the reviewer's last question, in 2021, the employment rate for women between 20-65 years was 79 percent.[14] These rates are derived from Sweden's annual statistics, which are

calculated at the end of the year based on employment status in November. As a result, small variations in employment rates may arise due to age range differences and timepoints when data was collected. Additionally, our employment status was self-reported, which is not entirely comparable to register data.

The online survey took place between May and September 2021- this was still a time highly impacted by the pandemic, for instance with larger proportion of people working from home. Please comment somewhere in the manuscript (discussion?) if/ how the timing of the survey may have affected the results. For instance, is working from home perhaps less demanding on pwMS's decreased energy, and therefore their relative need for adjustments and support from employers less marked than when working at the workplace?

Author's response: We thank the reviewer for this comment. Indeed, and as previously discussed with reviewer #2, the timing of the survey may have affected our results. These implications are included in the discussion section of the revised manuscript (page 10, paragraph 2).

At the time the survey was prepared and distributed, the COVID-19 outbreak had not yet peaked in Sweden. As a result, questions about the impact of COVID-19 were formulated more generally (e.g., whether participants had contracted COVID or if their employment had been affected by it). Unfortunately, we do not have more specific data to determine whether there was a reduced need for employer support due to participants working from home.

Register Variables. Page 6, lines 7-28.
Consider discussing the limitations of EDSS, for instance in regards to non-visible MS symptoms such as fatigue, depression, cognitive dysfunction. This may be relevant regarding a high percentage of your sample with mild MS severity however a large percentage of them report being affected by non-visible symptoms.

Author's response: We agree with the reviewer that the EDSS may not capture the full range of MS symptoms, particularly cognitive impairments and fatigue, as it primarily emphasizes physical mobility. To address this, we included additional clinical and symptoms variables in the regressions, such as SDMT and Fatigue scale from Neuro-Qol, to provide a more comprehensive assessment. The depression and anxiety subscales of Neuro-Qol were included in our preliminary analysis but are not included in the final version as it correlated heavily with each other (r=.80) and with fatigue (r=.54), introducing multicollinearity issues in the regression models. This information is now introduced in the methods section (page 6, statistical analysis, paragraph 2).

Survey measures. Page 6, lines 59-60.
"Are you satisfied with your treatment?" is a very open-ended question. Somewhere else in the manuscript (page 10, lines 49-50) the authors mention the hypothesis that subjects possibly answered this thinking of their use of DMTs. I do not see data here to support this hypothesis. Treatment satisfaction can be influenced by multiple factors: Satisfaction with their clinicians; satisfaction with the support staff; with access to timely appointments; cost of care; effectiveness of medication; side effects; their perceived severity of their MS or their perceived disability can impact negative perception about 2 treatment. It can also be affected by depression (lifetime prevalence in MS 25-50%), which can result in negative perceptions about several variables including this one.

Author's response: This was a closed-ended question with three possible responses: "yes," "somewhat," or "no." It followed a prior question asking, "*What treatment(s) are you currently receiving for your MS?*" and was accompanied by an open-ended question, *"Please explain your answer."* Most respondents commented on the drug's effects or the convenience of the treatment's frequency and method of administration. Hence, while we agree with the reviewer that treatment satisfaction can be influences by multiple factors, we believe the only valid interpretation of treatment satisfaction in this specific question is related to the therapy itself (as it followed a specific question on current treatment). We apologize if the lack of information might have caused this confusion. We have amended this in the revised manuscript in the methods (page 6, lines 86-90).

Results:
Page 7 Lines 50-51: While 53.5% of respondents were not married/not in partnership, 60.4% had
children living at home. Are these percentages comparable to Sweden's general female population?
Please disregard if they are comparable- I am curious about a higher percentage of non-partnered women due to their MS.

Author's response: We hypothesize that the younger age of the participants could lead to a higher percentage of number of children compared to the typical statistics of working age range (20-65), where individuals over 18 are no longer considered living at home but rather classified as single. Additionally, there may be a contemporary trend toward lower marriage rates or higher rates of separation/divorce, which would also fall under the category of not married or cohabiting.

In one of our earlier studies utilizing data from the Swedish MS register and the general population registered in Sweden, we matched PwMS were matched to a reference group at a ratio of 1:10 based on age, gender, living area, and county. We found that the percentages of those married/partnership (61.6%) and those single (38.3%) differed from the rates reported by Survey respondents in our study. These rates are not entirely comparable to Sweden's general female population; however, these differences may be attributed to the earlier study, which used data from 2009. Nevertheless, the findings showed that women with MS had a slightly higher percentage of non-partnership compared to the reference group.[13] Additionally, family composition differed significantly regarding the presence of children at home. PwMS had higher percentages of being single without children but lower rates of being single with children at home. These patterns were consistent when comparing women with and without MS.[13]

Lines 53-54: Cut off of 50y/o and excluding non-working women could have contributed to this sample presenting high percentage of RRMS with low EDSS.

Author's response: Yes, this limitation has been introduced in the revised version of the manuscript (page 10, paragraph 2).

Page 8 line 14: There seems to be something missing in that sentence. For instance ...had a positive association with HRQoL ***and*** were associated with higher HRQoL.(?)

Author's response: We thank the reviewer for bringing our attention to this omission. It has now been corrected in the revised manuscript.

Broadly speaking, it seems that (as expected, and previously reported): Worse clinical profile is

associated with worse HRQoL; milder clinical profile is associated with better HRQoL. Please list more clearly / separate clearly your findings regarding the other variables (satisfaction with each type of support; satisfaction with treatment) and their association with HRQoL once MS severity/ illness factors are controlled for. Please see the first paragraph below, I feel that further clarification in the results section may be also needed regarding the impact of demographic factors once clinical factors are controlled for.

Author's response: Following the reviewer's suggestions, some modifications in this respect have been included in the manuscript (abstract; results section: page 7, paragraph 3; discussion section, page 8, first paragraph) and removed from conclusion paragraph (page 11).

General, for Results: The "Invisible symptoms" include many problems highly prevalent in MS. In addition to fatigue and cognitive difficulties, pain and depression (and anxiety). I am curious if you have access to pain scores for this sample, or depression/ anxiety (emotional distress) scores to disentangle this variable.

Author's response: We have information regarding the anxiety and depression levels, which were both significant for those with visible and invisible limiting symptoms. However, when studying all clinical scales and symptoms together, fatigue and EDSS were the only factors remaining significative among women with MS having invisible symptoms as the most limiting symptom. In our preliminary analysis, correlation matrices showed how the subscales of Neuro-QoL correlated so strongly to each other (anxiety & depression with fatigue subscales). Hence, we excluded these variables from the main models and studied only the fatigue levels. We have included a comment on this regard in the revised manuscript (methods section; page 6, second paragraph).

Discussion.

Page 9, lines 10-11: "our findings suggest that HRQoL ...can be determined by demographic. ..characteristics". Please clarify. The way this statement reads, it places demographic characteristics at the same level as disease-related characteristics. However in the Results: it seems that demographic characteristics become less important once adjusted for other factors (Page 8, lines 13 and on: "However, when all key factors are considered...". This is also mentioned in Discussion, page 9 line 44. Please clarify. I think a more accurate way to discuss your findings may be that demographic factors at large were NOT associated w HRQoL once other variables were controlled (except for one or two exceptions).

Author's response: We thank the reviewer for this remark. Following previous comments from the reviewer, we have made changes regarding the description of these results as well as lowered the level of influence of the demographic characteristics in the abstract, results and discussion section.

Page 9, lines 18-19. I am not sure I would clump together "treatment" with the other two types of
perceived supports. As discussed previously satisfaction with treatment may involve a number of factors including biological effectiveness. We are talking about the perception not the effect but yes, it is ambiguous.

Author's response: We understand the reviewer's concern; however, as previously mentioned, the treatment satisfaction question was designed as a follow-up to the type of treatment participants were currently using. Nearly all participants responded by specifying the name of the drug they were taking. Although this information was not used in the study (we used registered data instead for ongoing treatment). However, while we cannot be certain how

participants interpreted the question, we believe the majority based their responses on their perceived experience of how the treatment was working for them.

Unfortunately, we could not find a better word choice than "treatment satisfaction" to describe this dimension, viewing it as a key component within the broader set of factors affecting the patient's daily life. However, we are open to any suggestions for a clearer and less ambiguous term to name this category.

Page 9 line 26: women handle MS impact more effectively than men: this is interesting, but it is not studied (duplicated or not) here.

Author's response: We thank the reviewer for pointing out this sentence which has now been removed from the discussion section.

Page 9 lines 27-28: I would distinguish clinical factors and symptoms (MS severity, fatigue etc) from "domains of life" such as support; would not use the same collective noun for all. This is semantics, does not affect the findings.

Author's response: We thank the reviewer for this suggestion. Changes have been made accordingly.

Page 9 lines 28-29: "MS severity (focused on physical disability)": Consider commenting on the limitation that because you used a cutoff of age 50 and because you excluded female respondents who are not working, you may have captures a less disabled sample therefore this aspect of MS was not as significantly included in this study.

Author's response: This has now been included as a limitation in the reviewed manuscript (page 10, paragraph 2).

Page 10 lines 9-11: As discussed before, I am curious how clear the influence of satisfaction with perceived support is, after controlling for disease severity.

Author's response: Associations between treatment satisfaction levels and HRQoL did not variate after controlling for disease severity (EDSS). This behaviour also applies to the dimensions of support from work and support from family and friends.

Page 10 lines 26-27: Please briefly comment on the legislation in Sweden regarding employers' obligation to provide support once people disclose an illness. Related to this, please also comment on percentage of people in Sweden who disclose their illnesses at work.

Author's response: The cornerstone of Swedish disability policy is the principle that everyone is of equal value and has equal rights. Sweden has ratified the CRPD and implemented the EU directive 2000/78/EC on discrimination, covering equal treatment and reasonable accommodation by employers, though case law on adjustments is still undeveloped. Positive measures include financing workplace adjustments and supported employment, but Sweden does not have a disability quota scheme.[15] Following, employers in Sweden can receive financial support from the Swedish Public Employment Service (Arbetsförmedlingen) to help employees with disabilities perform their work. [16] This support includes adapting the workplace to accommodate various disabilities, provided the employee has been with the company for no more than 12 months. Depending on the specific needs, such as assistive devices or modifications to the premises, either the employee or the employer can apply for

these allowances. According to another study of ours based on the same survey data, 79% of working PwMS choose to disclose their illness at work. [17] Our findings suggested that disclosure is less likely amongst PwMS experiencing "invisible symptoms" like fatigue or cognitive difficulties than physical disabilities. Moreover, receiving work-related adjustments is reliant upon a supportive work environment and often necessitates disclosure.

Page 10 lines 33-34: The invisible limitations of MS include other problems that are also (as fatigue is) very prevalent in MS- including pain, depression, and cognitive dysfunction. Please consider editing this to include these other phenomena.

Author's response: We agree with the reviewer that the invisible limitations of MS include much more than fatigue or cognitive dysfunction. As previously commented, we had information regarding anxiety and depression as subscales of the Neuro-QoL variables. However, these variables were not included in the final analysis given high correlation with each other and multicollinearity issues in the regression models when introduced together with the fatigue scales. We have included a comment on this regard in the revised manuscript (methods section; page 6, second paragraph).

Page 10 line 49: as I mentioned above, "treatment satisfaction" can include a large number of aspects. Not sure how we know here that respondents were thinking of DMTs when they answered; we did not ask them.

Author's response: We have made changes in the manuscript to clarify this treatment satisfaction statement was a follow-up question related to their current therapy (DMTs in almost all cases) which was cross-checked with register data ("ongoing treatment").

References:

1. McKay KA, Ernstsson O, Manouchehrinia A, Olsson T, Hillert J (2020) Determinants of quality of life in pediatric- and adult-onset multiple sclerosis. Neurology 94:e932–e941

2. Mitchell AJ, Benito-León J, González J-MM, Rivera-Navarro J (2005) Quality of life and its assessment in multiple sclerosis: integrating physical and psychological components of wellbeing. Lancet Neurol 4:556–566

3. Hemmett L, Holmes J, Barnes M, Russell N (2004) What drives quality of life in multiple sclerosis? QJM: An International Journal of Medicine 97:671–676

4. Murley C, Dervish J, Machado A, Svärd V, Wennman-Larsen A, Hillert J, Friberg E (2024) Exploring the working life of people with multiple sclerosis during the COVID-19 pandemic in Sweden. BMC Public Health 24:1389

5. Murley C, Pettersson E, Hillert J, Machado A, Friberg E (2024) Coronavirus disease 2019 infection among working-aged people with multiple sclerosis and the impact of disease-modifying therapies. Mult Scler J Exp Transl Clin 10:20552173241248293

6. Aksoy O, Wu AF-W, Aksoy S, Rivas C (2024) Social support and mental well-being among people with and without chronic illness during the Covid-19 pandemic: evidence from the longitudinal UCL covid survey. BMC Psychology 12:136

7. Papa A, Koutelekos I, Stefanidou S, Chrysovitsanou C, Polikandrioti M (2021) Factors associated with perceived social support of patients with multiple sclerosis. Curr J Neurol 20:64–72

8. Statistik för vaccination mot covid-19 — Folkhälsomyndigheten. https://www.folkhalsomyndigheten.se/folkhalsorapportering-statistik/statistikdatabaser-och-visualisering/vaccinationsstatistik/statistik-for-vaccination-mot-covid-19. Accessed 23 Sep 2024

9. Strålin K, Walther S, Holm J, Wahlström J, Bennet BArk A, Huergren M, Lindén T, Hanberger H (2020) National Board of Health and Welfare (Socialstyrelen). Nation-wide study of covid-19 care in Swedish hospitals – 81 % discharged alive.

10. Iacobaeus E, Burman J, Landtblom A-M (2020) [Not Available]. Lakartidningen 117:20129

11. Försäkringskassan (2024) Coronaviruset – det här gäller. https://www.forsakringskassan.se/privatperson/coronaviruset-det-har-galler. Accessed 23 Sep 2024

12. Lehmann AI, Rodgers S, Kamm CP, et al (2020) Factors associated with employment and expected work retention among persons with multiple sclerosis: findings of a cross-sectional citizen science study. Journal of Neurology 267:3069–3082

13. Machado A, Azad A, Pettersson E, Hillert J, Alexanderson K, Friberg E (2023) Gender differences in earnings among people with multiple sclerosis and associations with type of occupation and family composition: A population-based study with matched references. PLOS ONE 18:e0288998

14. Population by Labour market status. https://www.scb.se/am0210-en. Accessed 23 Sep 2024

15. The National board of Health and Welfare [Socialstyrelsen] Swedish disability policy – service and care for people with functional impairments.

16. Bidrag till hjälpmedel på arbetsplatsen. In: Arbetsförmedlingen. https://arbetsformedlingen.se/other-languages/english-engelska/additional-support/support-a-z/bidrag-till-hjalpmedel-pa-arbetsplatsen. Accessed 23 Sep 2024

17. Machado A, Murley C, Dervish J, Teni FS, Friberg E (2023) Work Adjustments by Types of Occupations Amongst People with Multiple Sclerosis: A Survey Study. J Occup Rehabil. https://doi.org/10.1007/s10926-023-10142-2

VERSION 2 - REVIEW

Reviewer **1**
Name **O'Shea, Dede**
Affiliation **UMass Chan**
Date **23-Oct-2024**
COI

Thank you to the authors for addressing previous comments. I find the responses to my comments and improvements to the manuscript to be adequate, in particular clarifying aspects of the methods and limitations of the study.

Reviewer **2**
Name **Mustač, Filip**
Affiliation **University Clinical Hospital Center Zagreb, Department of Psychiatry and Psychological Medicine**
Date **21-Oct-2024**
COI

The authors meaningfully explained what was asked of them in the review. In this form, the work is more suitable for publication. With that in mind, I have no further questions for the authors regarding this manuscript, which can be useful for the wider professional and scientific public.

Reviewer **3**

**Name**	**Safar, Laura**
**Affiliation**	**Harvard Medical School, Psychiatry**
**Date**	**15-Nov-2024**
**COI**	

Thank you for addressing my comments and questions.
